# Fibroblast Growth Factor 19 and Fibroblast Growth Factor 21 Regulation in Obese Diabetics, and Non-Alcoholic Fatty Liver Disease after Gastric Bypass

**DOI:** 10.3390/nu14030645

**Published:** 2022-02-02

**Authors:** Jiun-Yu Guo, Hsin-Hung Chen, Wei-Jei Lee, Shu-Chun Chen, Shou-Dong Lee, Chih-Yen Chen

**Affiliations:** 1 Division of Cardiology, Department of Medicine, Taipei Veterans General Hospital, Taipei 112201, Taiwan; jyguo@vghtpe.gov.tw; 2 Department of Nutrition and Health Sciences, Chang Jung Christian University, Tainan 71101, Taiwan; hsinhung@mail.cjcu.edu.tw; 3 Department of Surgery, Min-Sheng General Hospital, Taoyuan 330056, Taiwan; wjlee_obessurg_tw@yahoo.com.tw; 4 Department of Nursing, Chang-Gung Institute of Technology, Taoyuan 33303, Taiwan; N002916@e-ms.com.tw; 5 Division of Gastroenterology, Department of Internal Medicine, Cheng-Hsin General Hospital, Taipei 11220, Taiwan; ch9318@chgh.org.tw; 6 Division of Gastroenterology and Hepatology, Department of Medicine, Taipei Veterans General Hospital, Taipei 112201, Taiwan; 7 Faculty of Medicine and Institute of Emergency and Critical Medicine, College of Medicine, National Yang Ming Chiao Tung University, Taipei 11221, Taiwan; 8 Chinese Taipei Society for the Study of Obesity, Taipei 110301, Taiwan; 9 Taiwan Association for the Study of Small Intestinal Diseases, Taoyuan 333423, Taiwan

**Keywords:** obesity, diabetes mellitus, FGF 19, FGF 21, total bile acid, non-alcoholic fatty liver disease, gastric bypass

## Abstract

Background: Gastric bypass (GB) is an effective treatment for those who are morbidly obese with coexisting type 2 diabetes mellitus (T2DM) or non-alcoholic fatty liver disease (NAFLD). Fibroblast growth factors (FGFs) are involved in the regulation of energy metabolism. Methods: We investigated the roles of FGF 19, FGF 21, and total bile acid among those with morbidly obese and T2DM undergoing GB. A total of 35 patients were enrolled. Plasma FGF 19, FGF 21, and total bile acid levels were measured before surgery (M0), 3 months (M3), and 12 months (M12) after surgery, while the hepatic steatosis index (HSI) was calculated before and after surgery. Results: Obese patients with T2DM after GB presented with increased serum FGF 19 levels (*p* = 0.024) and decreased total bile acid (*p* = 0.01) and FGF 21 levels (*p* = 0.005). DM complete remitters had a higher FGF 19 level at M3 (*p* = 0.004) compared with DM non-complete remitters. Fatty liver improvers tended to have lower FGF 21 (*p* = 0.05) compared with non-improvers at M12. Conclusion: Changes in FGF 19 and FGF 21 play differential roles in DM remission and NAFLD improvement for patients after GB. Early increases in serum FGF 19 levels may predict complete remission of T2DM, while a decline in serum FGF 21 levels may reflect the improvement of NAFLD after GB.

## 1. Introduction

Obesity has been a global concern for the past 50 years and the prevalence has increased significantly over the past decade [1]. Obesity represents a major health challenge because it substantially increases the risk of metabolic diseases, including type 2 diabetes mellitus (T2DM) and non-alcoholic fatty liver disease (NAFLD) [1,2].

Body weight reduction is an important approach in reducing insulin resistance and improving NAFLD. Weight loss has been shown as one of the strongest predictors of improved insulin sensitivity [3]. The magnitude of weight loss is also correlated with the improvement of NAFLD [4].

Surgical intervention is considered an important approach, especially for morbidly obese patients with T2DM, medically resistant arterial hypertension, or comorbidities that are expected to improve with weight loss [5]. Gastric bypass, a widely adapted surgical technique, is one of the most effective methods to combat obesity and remit T2DM [6]. Nevertheless, there are many patients whose DM and NAFLD fail to improve despite receiving these interventions [7]. Furthermore, the mechanisms by which it causes weight loss and T2DM and NAFLD resolutions are not well elucidated [8]. 

Numerous studies have attempted to identify robust biological and clinical predictors of DM remission after bariatric surgery [9,10]. On the other hand, studies for improving NAFLD are relatively lacking and mostly limited to animal studies [11]. More biomarkers from the blood as surrogates in the evaluation of NAFLD to replace paired liver biopsy and reduce the suffering of the patients are desperately needed [12].

The human fibroblast growth factor (FGF) family contains at least 22 members involving the biological processes of cell growth, differentiation, development, and metabolism [13]. Aside from most FGFs presenting functions as autocrine or paracrine factors, FGF 19, FGF 21, and FGF 23 lack the conventional FGF heparin-binding domain and possess the ability to elicit endocrine actions, functioning as hormones [13]. Emerging evidence demonstrates the potential role of the FGF family in energy metabolism and in counteracting obesity, especially FGF 19 and FGF 21 [14]. Animal studies have shown that overexpression of FGF 19 or FGF 21 or treatment with recombinant protein enhanced metabolic rates and decreased fat mass, in addition to demonstrating improvements in glucose metabolism, insulin sensitivity, and lipid profiles [15,16,17,18].

Bile acids have a significant relationship with energy balance. Farnesoid-X-receptor (FXR) regulates bile acid homeostasis by regulating the transcription of several enterohepatic genes. The activation of the transcription factor FXR by bile acids provokes the subsequent secretion of FGF 19 [19]. Human FGF 19 is expressed in the ileal enterocytes of the small intestine. FGF 19, secreted into the portal circulation, has a pronounced diurnal rhythm, with peaks occurring 90–120 min after serum bile acid levels after food intake. β-Klotho (KLB) works as a co-receptor and supports endocrine signaling via binding with FGF receptor (FGFr) 4 [20]. The binding of FGF 19 to the hepatocyte cell surface FGFr 4/KLB complex leads to negative feedback and reduces hepatic bile salt synthesis [21]. Mice with impaired function for gut secretion of FGF 19 show significantly impaired weight loss and glucose improvement following bariatric surgery [15]. Collective data also reveal that the serum FGF 19 levels are decreased in patients with T2DM [22].

Apart from FGF 19, FGF 21 is expressed in multiple tissues, including the liver, brown adipose tissue, white adipose tissue, and pancreas [23]. Under normal physiologic status, most circulating FGF 21 originates from the liver [24]. Secretion of FGF 21 is provoked significantly by excess food intake, ketogenic, high-carbohydrate diets, or protein restriction [25]. The expression of the *FGF 21* gene depends on several pathways. Increased circulating free fatty acids and prolonged fasting promote the transcriptional activation of FGF 21 by the peroxisome proliferator-activated receptor α-mediated pathway [26,27]. A high-glucose diet activates carbohydrate-response element-binding protein (ChREBP) and enhances FGF 21 secretion [28]. Furthermore, general control nonderepressible 2 (GCN2) would be activated when encountering amino acid deficiency, leading to *FGF 21* transcription [29]. Metabolic stresses such as obesity, T2DM, or NAFLD are also responsible for inducing the expression and/or signaling of FGF 21 [30]. The FGF 21-dependent signaling of downstream FGFr is extremely complicated and well-debated [31]. Based on evidence from a recent study, FGF 21 stimulates hepatic fatty acid oxidation, ketogenesis, and gluconeogenesis, and suppresses lipogenesis [25]. FGF 21 reduced plasma glucose and triglycerides to a nearly normal level in an animal model [32].

Although recent studies provide clues regarding the dynamics of FGF 19 and FGF 21 in patients receiving bariatric surgery [8,33], the information is limited to sleeve gastrectomy [8]. Moreover, different characteristics between those with and without the improvement of obesity-related comorbidities were also lacking [33].

The main purpose of our study was to evaluate the effect of GB on changes in serum FGF 19 and FGF 21 levels. Furthermore, we also determined the relationship between both blood biomarkers and the improvement of either T2DM or NAFLD.

## 2. Materials and Methods

### 2.1. Study Design and Patients

This was a hospital-based prospective observational study. Obese patients with T2DM receiving GB surgery were enrolled in the study. The study was conducted in accordance with the Declaration of Helsinki and the study protocol was approved by the institutional review board (approval number: MSIRB2015017, YM 103127E, 201002037IC, 2011-09-015IC, CHGH-IRB (405)102A-52)).

The inclusion criteria were as follows: (1) been diagnosed with T2DM for more than 6 months with a baseline hemoglobin A1c (HbA1c) level > 8%; (2) receiving regular medical treatment for T2DM, including therapeutic nutritional therapy, oral anti-diabetic agents, or insulin under the evaluation of specialists; (2) body mass index (BMI) ≥ 32.5 kg/m^2^; (3) willingness to receive additional treatment, including diet control and lifestyle modifications; (4) willingness to accept follow-up appointment, and provided the informed consent documents.

We excluded patients who had the following diagnosis or baseline characteristics: (1) cancer within the previous 5 years; (2) human immunodeficiency virus infection or active pulmonary tuberculosis; (3) unstable cardiovascular condition within the past 6 months; (4) pulmonary embolisms; (5) serum creatinine levels > 2.0 mg/dL; (6) chronic hepatitis B or C, liver cirrhosis, inflammatory bowel disease, or acromegaly; (7) history of organ transplantation or other bariatric surgery; (8) alcohol use disorders or substance use disorder; or (9) those who were uncooperative.

All study patients returned for a monthly follow-up appointment after surgery. The patients received a face-to-face consultation with dietitians and reported a food diary log. Clinical anthropometry and laboratory assessments were performed simultaneously.

### 2.2. Surgical Technique of Gastric Bypass (GB)

GB was performed as described in our previous studies [34,35,36]. A five-port technique was used. The dissection begins directly on the lesser curvature, and a 15- to 20-mL gastric pouch is created using multiple EndoGIA-45 staplers (US Surgical Corp). Patients are then placed in a neutral position for the creation of the jejunostomy. The jejunum is divided 50 cm distal to the ligament of Treitz. A stapled end-to-side jejunojejunostomy is performed with a 100 cm Roux limb. The enteroenterostomy defect and all mesenteric defects are closed with continuous sutures. The Roux limb is tunneled via a retrocolic, retrogastric path and positioned near the transected gastric pouch. The CEEA-21 anvil (US Surgical Corp) is pulled into the gastric pouch transorally following the previous study [37]. The CEEA stapler is then inserted through the Roux limb to perform the end-to-side gastro-jejunostomy. After the test for air leak, the trocar fascial defects are closed. Two hemovac drains are left in the lesser sac and left subphrenic space separately [38].

### 2.3. Metabolic Profiles and Blood Sampling

All study subjects received clinical anthropometry and laboratory assessments on the day before surgery as baseline (M0) and at 3 months (M3) and 12 months (M12) after surgery. Metabolic profiles included anthropometry measurements (body weight, waist circumference, body mass index (BMI), and a body shape index (ABSI)), and systolic and diastolic blood pressure. The patients were required to fast overnight before each blood sample collection. Blood samples were obtained from the median cubital vein between 8 and 11 o’clock in the morning and immediately transferred into a chilled glass tube containing disodium EDTA (1 mg/mL) and aprotinin (500 units/mL). The samples were stored on ice during collection. They were further centrifuged, plasma-separated, aliquoted into polypropylene tubes, and stored at −20 °C before receiving analysis.

Laboratory assessments included serum levels of total cholesterol, triglycerides, high-density lipoprotein cholesterol (HDL-C), low-density lipoprotein cholesterol (LDL-C), fasting blood sugar, hemoglobin A1c (HbA1c), c-peptide, insulin, creatinine, uric acid, and liver function test (alanine transaminase (ALT), aspartate transaminase (AST), alkaline phosphatase (Alk-p), and gamma-glutamyl transferase (γ-GT)). All data are reported as means ± standard deviations.

### 2.4. Measurement of the Plasma FGF 19, FGF 21, and Serum Total Bile Acid Levels

The fasting blood samples obtained were used to determine the plasma levels of FGF 19 and FGF 21, and serum levels of total bile acids. Enzyme immunoassays for plasma FGF 19 and FGF 21 (R&D Systems, Minneapolis, MN, USA) were performed. Fasting total serum bile acids were assayed using the 3α-hydroxysteroid dehydrogenase method (Fumouze Diagnostics, Levallois-Perret, France). All measurements were performed in duplicate.

### 2.5. Definition of DM Complete Remission and Insulin Resistance

In our study, DM complete remission is defined as a return to normal measures of glucose metabolism with HbA1c in the normal range (<6.0% (42 mmol/mol)), which was adopted in most previous investigations [39]. We define HbA1c < 6.0% after 12 months after GB as DM complete remitters.

Insulin resistance was evaluated by using the homeostasis model assessment of insulin resistance (HOMA-IR), defined as fasting plasma glucose level (mmol/L) × fasting immunoreactive insulin level (µ U/mL)/22.5. The β-cell function was assessed using the homeostatic model assessment of β-cell function (HOMA-β), with the formula of [20 × fasting immunoreactive insulin level (µ U/mL)]/[fasting plasma glucose level (mmol/L) − 3.5] [40]. The variation of the area under the curve of plasma FGF 19 levels per minute (ΔAUC of plasma amylin) during the OGTT was calculated with the trapezoidal method [41].

### 2.6. Definition of NAFLD Based on the Hepatic Steatosis Index (HSI)

NAFLD has been considered as a continuum from obesity to metabolic syndrome and diabetes [42]. The gold standard to evaluate the magnitude of NAFLD depends on paired liver biopsy. However, liver biopsy may lead to patient discomfort and several complications, including bleeding, organ trauma, and even patient mortality [43]. Therefore, this approach is less feasible in clinical practice due to patient safety and a high patient refusal rate [12,44]. The hepatic steatosis index (HSI) was adapted for the clinical assessment of NAFLD and has been validated with imaging, including ultrasonography and magnetic resonance imaging [45]. HSI was defined as 8 × (ALT/AST ratio) + BMI (+2, if female; +2, if diabetes mellitus). A cut-off value of HSI > 36.0 may determine NAFLD with a specificity of 92.4% [46]. Based on this concept, our study defines HSI < 36.0 at 12 months after GB as HSI improvers (HSI-I).

### 2.7. Statistical Analysis

SAS (version 9.4) was applied for the data analyses. The paired *t*-test was used to compare preoperative age, BMI, waist circumference, total body fat percentage, and resting metabolic rate among patients who underwent GB between the preoperative period and 3 and 12 months after surgery.

Postoperative serum biochemical data (except BMI) were analyzed using an ANCOVA model to adjust for preoperative age and gender. The paired Student *t*-test was used in comparisons between preoperative data with data at each time point at 3 or 12 months postoperatively.

A trend analysis (to obtain *p* values for the trend) was performed using the repeated GLM to explore whether GB had a significantly different effect on postoperative indicators within 3 or 12 months postoperatively.

The correlations among different postoperative indicators to explain possible physiological pathways were evaluated using the Pearson correlation. A *p*-value of < 0.05 was considered statistically significant.

## 3. Results

### 3.1. Changes in Metabolic Profiles and Laboratory Data after GB

A total of 35 obese patients (12 males and 23 females) with T2DM who underwent GB were enrolled. The baseline average age, body weight, and BMI were 44.8 ± 9.7 years old, 84.8 ± 14.1 kg, and 31.6 ± 4.6 kg/m^2^, respectively. The duration of T2DM was 5.8 ± 4.9 years. All enrolled patients received GB and were followed up for more than 1 year subsequently.

Changes in metabolic profiles and laboratory data are reported in Table 1. Body weight, BMI, waist circumference, and ABSI showed significant improvements 1 year after GB (*p* < 0.001). Diabetes-related parameters, including fasting blood glucose, HbA1c, c-peptide, and insulin level, also showed a significant decline (*p* < 0.05). Liver function tests, including ALT, AST, and Alk-p levels, showed no significant change; however, a significant decline in γ-GT level was observed (*p* = 0.006). As for the lipid profile, HDL-C increased, and triglycerides decreased (*p* < 0.05), while total cholesterol and LDL levels did not change significantly. Decreases in uric acid levels were also demonstrated (*p* = 0.019).

**Figure 1:**
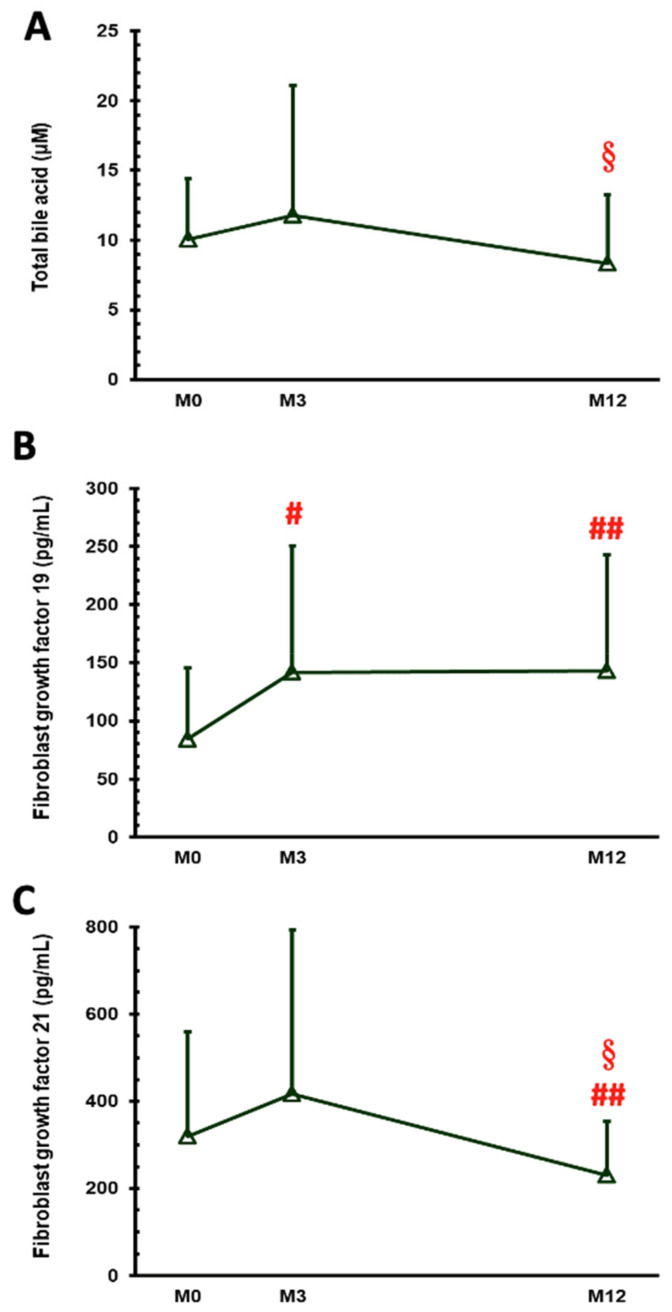
Serum levels of total bile acids (**A**), fibroblast growth factor 19 (**B**), and fibroblast growth factor 21 (**C**) in patients with obesity and type 2 diabetes before gastric bypass (M0) and 3 months (M3) and 1 year (M12) (B) after surgery. # *p* < 0.05, ## *p* < 0.01 compared with M0, and § *p* < 0.05 compared with M3.

Variations in serum levels among FGF 19, FGF 21, and total bile acids before the operation and after 3 months and 12 months are presented in Figure 1. All three markers showed a significant trend of change after GB (*p* = 0.024, 0.005, and 0.010, respectively). The total bile acid level changed from 10.07 ± 4.33 µM at M0 to 11.78 ± 9.32 µM at M3 and to 8.31 ± 4.95 µM at M12 (*p* = 0.023 between M3 and M12). The FGF 19 level increased from 84.20 ± 61.31 pg/mL at M0 to 141.76 ± 108.70 pg/mL at M3 and to 142.69 ± 100.21 pg/mL at M12 (*p* = 0.016 between M0 and M3, *p* = 0.002 between M0 and M12). The FGF 21 level changed from 320.06 ± 238.96 pg/mL at M0 to 416.99 ± 375.86 pg/mL at M3 and to 230.24 ± 123.71 pg/mL at M12 (*p* = 0.049 between M0 and M3, *p* = 0.005 between M0 and M12).

**Figure 2:**
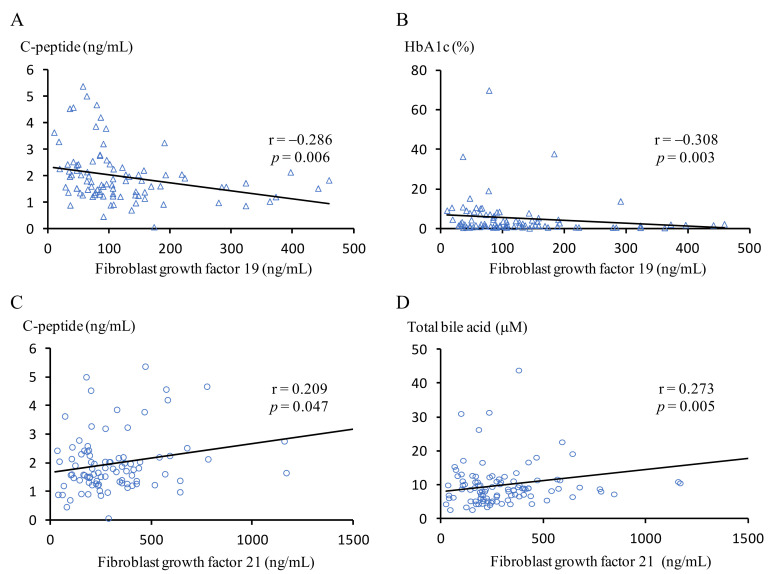
Relationships of the levels of fibroblast growth factor 19 with the levels of c-peptide (**A**) and HbA1c (**B**), and fibroblast growth factor 19 with c-peptide (**C**) and total bile acids (**D**), in patients with obesity and type 2 diabetes mellitus at M0, M3, and M12.

Serum levels of FGF 19 and FGF 21 for subjects during the three periods were intergraded and the correlations with indicators of diabetes and NAFLD are shown in Figure 2. FGF 19 had a significant negative correlation with serum c-peptide (r = −0.286, *p* = 0.006, Figure 2A) and HbA1c level (r = −0.308, *p* = 0.003, Figure 2B). On the other hand, FGF 21 had a significant positive correlation with serum HbA1c (r = 0.209, *p* = 0.047, Figure 2C) and total bile acids (r = 0.273, *p* = 0.005, Figure 2D), respectively.

### 3.2. Characteristic Differences between DM-CR and DM-Non-CR Subjects

Thirteen of our 35 study participants (37.1%) achieved complete DM remission 12 months after GB. Profiles of both DM-CR and DM-non-CR groups measured before and 12 months after GB are presented in Table 2.

Regarding the preoperative condition, the DM-CR group had higher baseline body weight (93.85 ± 16.25 vs. 79.43 ± 9.53 kg, *p* = 0.010), BMI (43.73 ± 5.00 vs. 29.79 ± 3.27 kg/m^2^, *p* = 0.001), waist circumference (108.69 ± 10.22 vs. 100.45 ± 9.13 cm, *p* = 0.020), c-peptide (3.23 ± 1.01 vs. 2.31 ± 1.18 mg/dL, *p* = 0.026), and ALT level (56.08 ± 40.45 vs. 33.05 ± 25.90 U/L, *p* = 0.047) but with lower baseline HbA1c (8.51 ± 1.42 vs. 9.76 ± 1.41%, *p* = 0.016) compared to the DM-non-CR group. In addition, the DM-CR group had higher HSI (51.30 ± 5.05 vs. 42.69 ± 4.75, *p* < 0.001). Baseline FGF 19, FGF 21, and total bile acid levels were similar between the two groups.

One year after the operation, the HbA1c level of the DM-CR group was 5.42 ± 0.38%, while that of the DM-non-CR group was 7.09 ± 0.99%. Patients who achieved DM-CR had lower fasting blood glucose (89.70 ± 10.22 vs. 125.18 ± 31.18 mg/dL, *p* < 0.001), γ-GT (12.40 ± 5.30 vs. 31.45 ± 24.71 U/L, *p* = 0.003), and triglyceride (74.00 ± 24.10 vs. 119.45 ± 46.79 mg/dL, *p* = 0.001) levels. No significant difference was found among other measurements regarding metabolic profiles or laboratory data. Neither FGF 19, FGF 21, nor total bile acid level showed any significant difference between DM-CR and DM-non-CR 12 months after GB.

**Figure 3:**
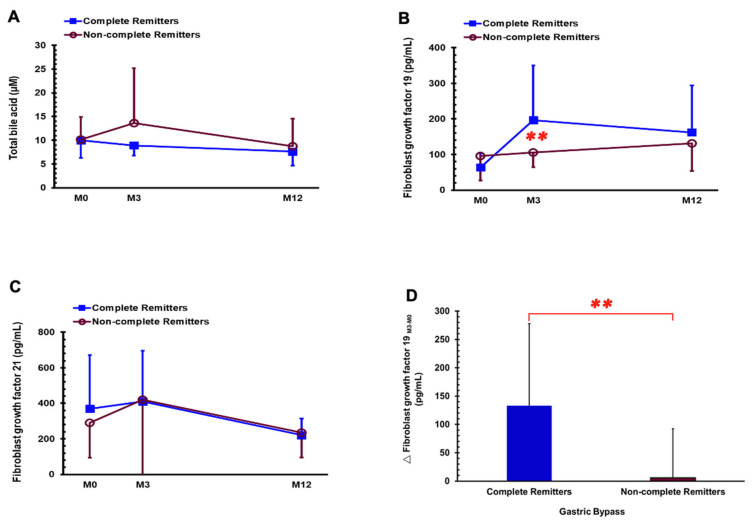
Serum levels of total bile acids (**A**), fibroblast growth factor 19 (**B**), fibroblast growth factor 21 (**C**) in the DM-CR and DM-non-CR groups before gastric bypass (M0) and 3 months (M3) and 1 year (M12) postoperatively and change in fibroblast growth factor 19 at 3 months after gastric bypass (**D**). ** *p* < 0.01 compared between the DM-CR and DM-non-CR groups after adjusted for age and gender.

Changes in serum levels of FGF 19, FGF 21, and total bile acids at the time before operation and 3 months and 12 months after surgery are presented in Figure 3. After adjustment for age and gender, the DM-CR group had a significantly higher FGF 19 level compared to DM-non-CR at M3 (196.88 ± 153.00 vs. 102.06 ± 34.72 pg/mL, *p* = 0.004, shown in Figure 3B), and more changes in FGF 19 between M3 and M0 (133.15 ± 144.65 vs. 6.15 ± 86.35 pg/mL, *p* = 0.001, shown in Figure 3D) compared with the DM-non-CR group.

### 3.3. Characteristic Differences between HSI-I and HSI-Non-I Subjects

Twenty-five of our 35 enrolled subjects (71.4%) were considered HSI-I based on evaluation at M12. Measurements of HSI-I and HSI-non-I groups before and at 12 months postoperatively are presented in Table 3.

Regarding the preoperative condition, patients among the HSI-I group had higher systolic blood pressure (139.60 ± 15.11 vs. 127.30 ± 10.14 mmHg, *p* = 0.024) and lower fasting blood glucose (161.44 ± 66.11 vs. 214.70 ± 70.31, *p* = 0.042). Neither baseline FGF 19, FGF 21, nor total bile acid level showed any significant difference between these two groups.

One year after GB, fatty liver improvers had an average HSI of 34.49 ± 1.25, while non-improvers had a HSI of 38.70 ± 1.93. HSI-I had lower serum FGF 21 (204.06 ± 122.68 vs. 295.67 ± 104.96, *p* = 0.046), insulin (3.43 ± 1.78 vs. 10.88 ± 10.38 mU/L, *p* = 0.0499), and triglyceride levels (90.18 ± 32.01 vs. 138.40 ± 55.67 mg/dL, *p* = 0.026). No differences in FGF 19 and bile acid levels were found.

**Figure 4:**
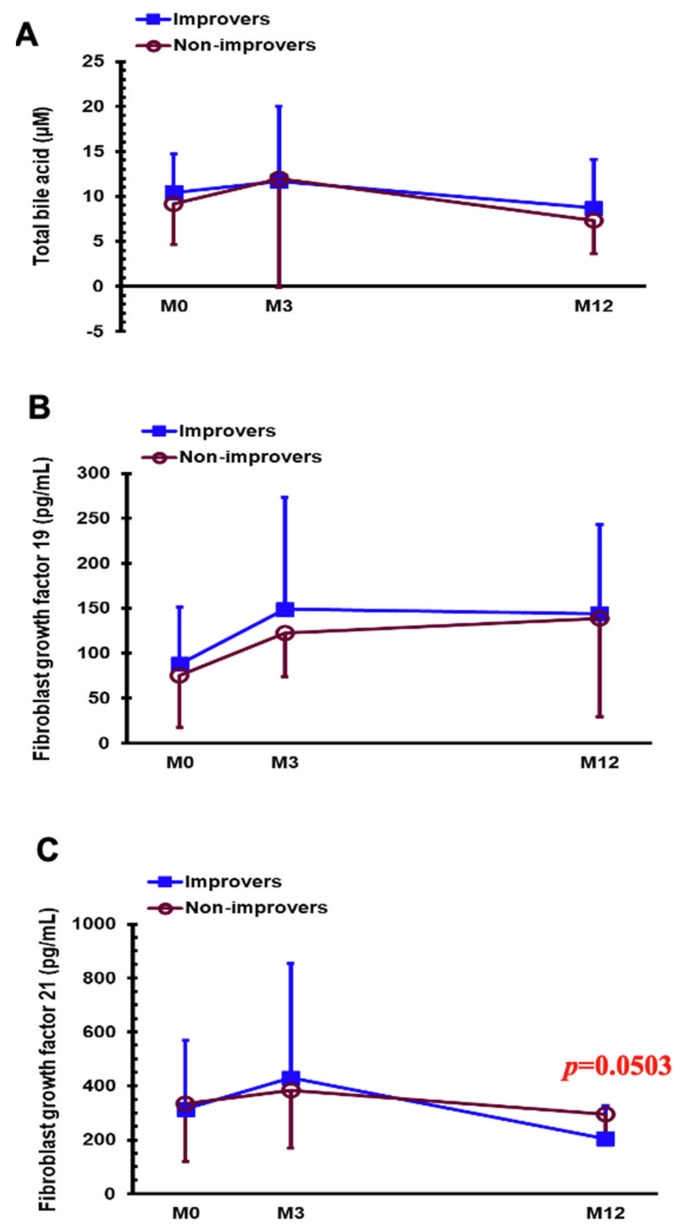
Serum levels of total bile acids (**A**), fibroblast growth factor 19 (**B**), and fibroblast growth factor 21 (**C**) in the HSI-I and HSI-non-I groups before gastric bypass (M0) and 3 months (M3) and 1 year (M12) postoperatively (*p* value evaluated after adjusted for age and gender).

The changes in FGF 19, FGF 21, and total bile acids are presented in Figure 4. The differences in FGF 21 between HSI-I and HSI-non-I showed borderline significance (*p* = 0.0503) after being adjusted for age and gender (Figure 4C).

## 4. Discussion

In our study, 35 obese patients with T2DM showed significant improvements in body weight, BMI, waist circumference, ABSI and insulin resistance, c-peptide, and insulin after receiving GB. Despite the limited effect in the improvement on lipid profile and liver function, our study also demonstrated GB as a beneficial approach to improving NAFLD based on the significant improvement of HSI after GB. Our study confirmed GB as an effective strategy to combat morbid obesity and its related comorbidities, such as T2DM and NAFLD.

Serum FGF 19 concentrations are consistently elevated in obese patients after GB. This finding is consistent with previous studies in patients receiving sleeve gastrectomy, another standard bariatric procedure [8,47]. FGF 19 has a close connection with obesity [47,48] and correlates negatively with BMI in obese patients with DM [22]. A recent meta-analysis demonstrated a negative association between FGF 19 levels and the degree of BMI reduction after bariatric surgery [49], while obesity and DM led to significantly lower FGF 19 levels compared to those without DM [48]. A recent study from Gómez-Ambrosi et al. [33] showed a subsequent increase in serum FGF 19 levels after either diet- or surgery-induced weight loss.

Another major purpose of our study was to provide additional information on the characteristics of FGF levels on DM remission. Our analysis showed a significant elevation of FGF 19 levels among DM remitters compared with non-remitters 3 months after GB. Early FGF 19 improvements may predict the complete remission of T2DM for obese patients receiving GB. Furthermore, our study pointed out a negative linear correlation between FGF 19 levels and the indicators of DM severity, including HbA1c and c-peptide levels. This implies that FGF 19 may also provide predictive value regarding improvements in insulin resistance and remission of T2DM.

FGF 21 signaling plays a crucial role in the development and progression of NAFLD [50]. Based on animal studies, the overexpression of FGF 21 antagonizes the effect of FGF 15/19 [51]. The elevation of FGF 21 levels in NAFLD patients may result from dysfunctional PPARα signaling [52]. Similar to the presence of insulin resistance in T2DM, “FGF 21 resistance” has been proposed as a key feature in NAFLD [53].

In one human study, FGF 21 had a positive correlation with BMI, and the expression of hepatic FGF 21 mRNA was significantly elevated in NAFLD [30]. It is an interesting fact, however, that the presence of FGF 21 elevation only presented during the stage of simple steatosis, and not after the development of non-alcoholic steatohepatitis or the resolution of steatosis [54]. This may be explained as reflecting the anti-inflammation effect of FGF 21 [55].

Our investigation showed that NAFLD improvers tended to have lower FGF 21 levels at 12 months after GB with borderline significance. This finding consisted of the effect of bariatric surgery on reducing hepatic fat, inflammation, and fibrosis [56]. Recent studies pointed out potential differences from the aspect of different surgical interventions [33,57]. The effect on changes in FGF 21 is more prominent in sleeve gastrectomy compared with GB [33]. Our previous instigation also showed a more prominent change in pancreatic polypeptide hormone after receiving sleeve gastrectomy [57]. The results from our current study agree with this finding and further comparison between different types of surgery with larger study populations may be required.

Despite FGF 19 and FGF 21 displaying some overlapping functions, our study showed differential roles of FGF 19 and FGF 21. FGF 19 had a higher affinity toward FGFr4, while FGF 21 is more potent toward FGFr1c. The FGFr4 gene is mainly expressed in the liver, whereas the FGFr1c gene has higher expression in the adipose tissue [58]. The biological evidence supports the fact that FGF 19 tends to have a closer interaction with DM and insulin resistance, while FGF 21 plays a greater role in the spectrum of fatty liver disease. Our study supports the notion that both FGF 19 and 21 may have complementary advantages in evaluating these two major comorbidities, T2DM and NAFLD, of obesity, respectively.

The understanding of these energy-regulating pathways suggests the potential of pharmacological approaches for patients with T2DM and NAFLD. Aldafermin (NGM 282), an engineered FGF 19 analog, demonstrated its safety and effectiveness in reducing liver fat content, improving liver fibrosis, and preventing the progression of non-alcoholic steatohepatitis (NASH) in a recent phase 2 randomized control trial [59], whereas its ability to improve insulin sensitivity remained inconsistent among studies [60]. Pegbelfermin and efruxifermin are considered the two most promising FGF 21 analogs. In a phase 2 study focusing on morbidly obese T2DM patients, high-dose pegbelfermin (BMS-986036), a PEGylated form of FGF 21, showed an effect on improving the lipid profile (increasing HDL-C and lower triglycerides), while it had no significant effect on improving weight loss or glycemic control [61]. Efruxifermin, a fusion protein of human IgG1 Fc domain linked to a modified human FGF 21, is considered a promising agent in reducing the liver fat fraction and markers of hepatic injury among NASH patients, as reported in a recent phase 2a study [62]. There is still limited evidence focusing on the obese population or on the aspect of combination or comparison with bariatric surgery.

Our study has several limitations. First, the sample size of our study is small and may neglect the potential differences in the two biomarkers while performing subgroup analysis. Second, the current diagnostic criteria of NAFLD depend on histological or image studies. Subjects in our study did not receive evaluations of NAFLD during enrollment, and we adapted HSI as a blood surrogate for non-invasive assessment. The HSI has been validated with other non-invasive approaches, including ultrasonographic and magnetic resonance imaging [46]. HSI is also widely adopted in the field of metabolic disorders [63,64].

## 5. Conclusions

Obesity represents a major health challenge in our modern society. GB is an effective surgical approach for weight loss, leading to an increase in FGF 19 levels and a decrease in total bile acids and FGF 21 levels postoperatively. FGF 19 levels had a negative correlation with the severity of T2DM based on c-peptide and HbA1c levels. FGF 21 levels had a positive correlation with c-peptide and total bile acid levels. Early increases in serum FGF 19 levels may be a predictor for complete remission of T2DM after GB. A decline in serum FGF 21 levels may reflect the improvement of NAFLD after GB. Our study may shed light on the differential roles of FGF 19 and FGF 21 in human T2DM remission and NAFLD improvement.

## Figures and Tables

**Table 1:** Characteristics of the obese patients with type 2 diabetes mellitus before gastric bypass (M0) and 3 months (M3) and 1 year (M12) postoperatively.

*n* = 35	M0	M3	M12	*p* Value
Metabolic profile				
Body weight (kg)	84.78 ± 14.12	69.64 ± 9.68	63.86 ± 6.75	<0.001
BMI (kg/m^2^)	31.63 ± 4.62	26.14 ± 3.19	24.41 ± 2.58	<0.001
Waist circumference (cm)	103.60 ± 10.25	90.40 ± 7.96	82.33 ± 5.30	<0.001
Excess weight loss (%)		19.00 ± 22.99	18.43 ± 10.83	0.346
ABSI	0.081 ± 0.005	0.077 ± 0.017	0.077 ± 0.004	<0.001
Systolic blood pressure (mmHg)	136.09 ± 14.84	132.54 ± 21.34	126.86 ± 16.31	0.076
Diastolic blood pressure (mmHg)	85.54 ± 10.73	81.23 ± 16.30	79.67 ± 14.15	0.277
Laboratory data				
Creatinine (mg/dL)	0.79 ± 0.32	0.76 ± 0.28	0.71 ± 0.29	<0.001
Fasting blood glucose (mg/dL)	176.66 ± 70.64	127.03 ± 46.69	114.09 ± 31.11	<0.001
HbA1c (%)	9.29 ± 1.52	7.07 ± 1.62	6.50 ± 1.16	<0.001
C-peptide (mg/dL)	2.65 ± 1.19	1.69 ± 0.59	1.39 ± 0.50	<0.001
Insulin (mU/L)	23.16 ± 28.90	7.10 ± 6.89	5.83 ± 6.86	0.007
HOMA-IR index	9.91 ± 13.39	2.03 ± 1.99	1.65 ± 2.02	<0.001
HOMA-β index	1.15 ± 1.95	0.54 ± 0.59	0.50 ± 0.67	0.060
ALT (U/L)	41.60 ± 33.46	35.43 ± 33.42	33.29 ± 33.20	0.251
AST (U/L)	32.17 ± 28.09	29.61 ± 20.95	28.90 ± 26.14	0.364
Alk-p (U/L)	61.46 ± 19.09	81.04 ± 40.46	69.81 ± 19.46	0.268
γ-GT (U/L)	42.29 ± 27.87	33.37 ± 42.45	25.10 ± 22.18	0.006
Total cholesterol (mg/dL)	193.60 ± 42.93	176.21 ± 34.03	168.09 ± 33.23	0.063
Triglyceride (mg/dL)	231.37 ± 217.51	127.36 ± 57.10	105.25 ± 45.93	0.006
HDL-C (mg/dL)	41.57 ± 7.80	37.93 ± 7.83	46.31 ± 10.23	<0.001
LDL-C (mg/dL)	117.89 ± 33.89	117.14 ± 31.26	105.69 ± 30.31	0.254
Uric acid (mg/dL)	5.64 ± 1.63	5.43 ± 1.46	5.11 ± 1.50	0.019
Total bile acid (µM)	10.07 ± 4.33	11.78 ± 9.32	8.31 ± 4.95	0.010
FGF 19 (pg/mL)	84.20 ± 61.31	141.76 ± 108.70	142.69 ± 100.21	0.024
FGF 21 (pg/mL)	320.06 ± 238.96	416.99 ± 375.86	230.24 ± 123.71	0.005
HSI	45.89 ± 6.39	38.96 ± 4.16	36.25 ± 2.61	<0.001

ABSI, a body shape index; BMI, body mass index; HbA1c, hemoglobin A1c; ALT, alanine aminotransferase; AST, aspartate transaminase; Alk-p, alkaline phosphatase; γ-GT, gamma-glutamyl transferase; HDL-C, high-density lipoprotein cholesterol; HOMA, homeostasis model assessment; IR, insulin resistance; LDL-C, low-density lipoprotein cholesterol; FGF, fibroblast growth factor; HSI, hepatic steatosis index.

**Table 2:** Characteristics of obese patients with type 2 diabetes mellitus before and 1 year after gastric bypass between the complete remitters of diabetes mellitus (DM-CR) and non-complete remitters of diabetes mellitus (DM-non-CR).

		M0			M12	
	DM-CR (*n* = 13)	DM-Non-CR (*n* = 22)	*p* Value	DM-CR (*n* = 13)	DM-Non-CR (*n* = 22)	*p* Value
Metabolic profile						
Body weight (kg)	93.85 ± 16.25	79.42 ± 9.53	0.010	62.6 ± 7.78	64.43 ± 6.38	0.511
BMI (kg/m^2^)	34.73 ± 5.00	29.79 ± 3.27	0.001	24.88 ± 4.08	24.20 ± 1.64	0.641
Waist circumference (cm)	108.69 ± 10.22	100.45 ± 9.13	0.020	80.56 ± 4.61	83.13 ± 5.51	0.234
ABSI	0.080 ± 0.006	0.082 ± 0.004	0.246	0.076 ± 0.006	0.078 ± 0.003	0.584
Systolic blood pressure (mmHg)	130.92 ± 13.45	139.14 ± 15.07	0.115	124.00 ± 20.70	128.00 ± 14.91	0.624
Diastolic blood pressure (mmHg)	83.54 ± 10.82	86.73 ± 10.75	0.404	75.33 ± 14.07	81.0 ± 14.29	0.389
Laboratory data						
Creatinine (mg/dL)	0.67 ± 0.15	0.86 ± 0.37	0.051	0.60 ± 0.11	0.76 ± 0.33	0.063
Fasting blood glucose (mg/dL)	164.69 ± 64.95	183.73 ± 74.34	0.449	89.70 ± 10.22	125.18 ± 31.18	<0.001
HbA1c (%)	8.51 ± 1.42	9.76 ± 1.41	0.016	5.42 ± 0.38	7.09 ± 0.99	<0.001
C-peptide (mg/dL)	3.23 ± 1.01	2.31 ± 1.18	0.026	1.35 ± 0.34	1.41 ± 0.57	0.791
Insulin (mU/L)	18.05 ± 9.24	26.19 ± 35.75	0.321	4.99 ± 3.07	6.24 ± 8.11	0.645
HOMA-IR index	7.21 ± 3.95	11.51 ± 16.55	0.256	1.06 ± 0.67	1.93 ± 2.38	0.152
HOMA-β index	0.81 ± 0.59	1.34 ± 2.41	0.331	0.61 ± 0.64	0.44 ± 0.69	0.479
ALT (U/L)	56.08 ± 40.45	33.05 ± 25.90	0.047	24.40 ± 17.56	37.52 ± 38.16	0.200
AST (U/L)	37.00 ± 27.61	29.32 ± 28.61	0.443	22.70 ± 12.05	31.86 ± 30.52	0.371
Alk-p (U/L)	64.46 ± 24.37	59.68 ± 15.55	0.482	62.70 ± 16.57	73.19 ± 20.18	0.164
γ-GT (U/L)	51.58 ± 34.04	36.42 ± 22.19	0.143	12.40 ± 5.30	31.45 ± 24.71	0.003
Total cholesterol (mg/dL)	186.62 ± 49.00	197.73 ± 39.54	0.468	155.50 ± 24.65	173.82 ± 35.50	0.151
Triglyceride (mg/dL)	179.08 ± 133.37	262.27 ± 252.47	0.212	74.00 ± 24.10	119.45 ± 46.79	0.001
HDL-C (mg/dL)	39.85 ± 7.71	42.59 ± 7.85	0.322	45.80 ± 7.94	46.55 ± 11.28	0.852
LDL-C (mg/dL)	119.46 ± 39.61	116.95 ± 31.00	0.836	95.10 ± 19.71	110.50 ± 33.34	0.187
Uric acid (mg/dL)	5.69 ± 1.58	5.61 ± 1.69	0.886	4.63 ± 1.18	5.33 ± 1.61	0.230
Total bile acid (µM)	9.97 ± 3.63	10.13 ± 4.77	0.919	7.61 ± 2.91	8.72 ± 5.86	0.462
FGF 19 (pg/mL)	63.73 ± 40.17	96.29 ± 68.93	0.131	162.41 ± 131.83	131.03 ± 77.10	0.445
FGF 21 (pg/mL)	370.14 ± 300.53	290.47 ± 195.89	0.348	221.17 ± 93.30	235.60 ± 140.44	0.744
HSI	51.30 ± 5.05	42.69 ± 4.75	<0.001	36.08 ± 3.10	36.33 ± 2.44	0.831

ABSI, a body shape index; BMI, body mass index; HbA1c, hemoglobin A1c; HOMA, homeostasis model assessment; IR, insulin resistance; ALT, alanine aminotransferase; AST, aspartate transaminase; Alk-p, alkaline phosphatase; γ-GT, gamma-glutamyl transferase; HDL-C, high-density lipoprotein cholesterol; LDL-C, low-density lipoprotein cholesterol; FGF, fibroblast growth factor; HSI, hepatic steatosis index.

**Table 3:** Characteristics of obese patients with type 2 diabetes mellitus before and 1 year after gastric bypass between the hepatic steatosis index improvers (HSI-I) and hepatic steatosis index non-improvers (HSI-non-I).

		M0			M12	
	HSI-I (*n* = 25)	HSI-Non-I (*n* = 10)	*p* Value	HSI-I (*n* = 25)	HSI-Non-I (*n* = 10)	*p* Value
Metabolic profile						
Body weight (kg)	86.19 ± 15.56	81.26 ± 9.38	0.358	62.42 ± 6.57	66.59 ± 6.55	0.116
BMI (kg/m^2^)	31.98 ± 5.14	30.73 ± 2.98	0.476	23.96 ± 2.78	25.27 ± 2.02	0.199
Waist circumference (cm)	104.04 ± 11.09	102.55 ± 8.31	0.705	81.11 ± 4.30	84.65 ± 6.43	0.087
ABSI	0.081 ± 0.005	0.082 ± 0.004	0.606	0.077 ± 0.004	0.077 ± 0.005	0.995
Systolic blood pressure (mmHg)	139.60 ± 15.11	127.30 ± 10.14	0.024	129.58 ± 15.08	123.22 ± 18.07	0.390
Diastolic blood pressure (mmHg)	87.00 ± 11.60	81.90 ± 7.46	0.209	80.92 ± 14.49	78.00 ± 14.37	0.652
Laboratory data						
Creatinine (mg/dL)	0.82 ± 0.36	0.72 ± 0.13	0.438	0.72 ± 0.35	0.68 ± 0.070	0.542
Fasting blood glucose (mg/dL)	161.44 ± 66.11	214.70 ± 70.31	0.042	108.91 ± 25.25	125.50 ± 40.45	0.166
HbA1c (%)	9.11 ± 1.70	9.76 ± 0.83	0.258	6.41 ± 1.11	6.72 ± 1.30	0.482
C-peptide (mg/dL)	2.51 ± 1.17	3.02 ± 1.22	0.255	1.27 ± 0.50	1.63 ± 0.44	0.062
Insulin (mU/L)	21.70 ± 24.92	26.82 ± 38.45	0.643	3.43 ± 1.78	10.88 ± 10.38	0.050
HOMA-IR index	7.94 ± 7.82	14.83 ± 21.82	0.353	0.81 ± 0.49	3.16 ± 2.80	0.027
HOMA-β index	1.33 ± 2.23	0.70 ± 0.88	0.397	0.33 ± 0.38	0.93 ± 1.01	0.098
ALT (U/L)	37.32 ± 32.73	52.30 ± 34.57	0.237	35.81 ± 39.21	28.00 ± 14.54	0.428
AST (U/L)	27.36 ± 21.08	44.20 ± 39.62	0.110	32.00 ± 33.78	22.40 ± 10.20	0.209
Alk-p (U/L)	64.20 ± 20.51	54.60 ± 13.49	0.183	70.33 ± 20.21	68.70 ± 18.79	0.831
γ-GT (U/L)	43.32 ± 31.22	39.78 ± 18.53	0.754	24.75 ± 19.72	25.80 ± 27.64	0.905
Total cholesterol (mg/dL)	193.40 ± 40.09	194.10 ± 51.72	0.966	168.18 ± 34.42	167.90 ± 32.25	0.983
Triglyceride (mg/dL)	189.52 ± 115.25	336.00 ± 355.37	0.231	90.18 ± 32.01	138.40 ± 55.67	0.026
HDL-C (mg/dL)	41.92 ± 7.04	40.70 ± 9.83	0.682	47.82 ± 9.13	43.00 ± 12.18	0.223
LDL-C (mg/dL)	122.44 ± 29.39	106.50 ± 42.84	0.214	105.36 ± 33.32	106.40 ± 23.94	0.930
Uric acid (mg/dL)	5.84 ± 1.57	5.13 ± 1.75	0.682	5.27 ± 1.67	4.77 ± 1.08	0.399
Total bile acid (µM)	10.43 ± 4.30	9.17 ± 4.50	0.444	8.71 ± 5.38	7.31 ± 3.71	0.457
FGF 19 (pg/mL)	87.83 ± 63.63	75.11 ± 57.23	0.587	144.15 ± 98.45	139.04 ± 109.87	0.894
FGF 21 (pg/mL)	314.54 ± 252.60	333.85 ± 212.77	0.833	204.06 ± 122.68	295.67 ± 104.96	0.046
HSI	46.29 ± 6.80	44.88 ± 5.42	0.562	34.49 ± 1.25	38.70 ± 1.93	<0.001

ABSI, a body shape index; BMI, body mass index; HbA1c, hemoglobin A1c; HOMA, homeostasis model assessment; IR, insulin resistance; ALT, alanine aminotransferase; AST, aspartate transaminase; Alk-p, alkaline phosphatase; γ-GT, gamma-glutamyl transferase; HDL-C, high-density lipoprotein cholesterol; LDL-C, low-density lipoprotein cholesterol; FGF, fibroblast growth factor; HSI, hepatic steatosis index.

## Data Availability

The dataset are available from the corresponding author at chency@vghtpe.gov.tw.
